# Supplementary material for: Influence of Ploidy and Genetic Background on Stress Tolerance of Intraspecific Yeast Hybrids
Source: Microb Biotechnol. 2026 Mar 25;19(3):e70337. doi: 10.1111/1751-7915.70337 (PMC13097363; doi:10.1111/1751-7915.70337)
Supplement: Supplementary file 1 — Data S1: mbt270337‐sup‐0001‐supinfo.zip. Table S1: Information about the wild‐type and mating‐competent yeast strains used in the study. Table S2: Information about the intraspecific yeast hybrids generated in this study. Table S3: Oligonucleotides used in this study. Table S4: The growth data collected in this study. [file MBT2-19-e70337-s001.zip › Supplementary_Table_Legends.pdf]

Supplementary Table Legends

Supplementary Table S1. Information about the wild-type and mating-competent yeast strains used in the study.

Supplementary Table S2. Information about the intraspecific yeast hybrids generated in this study.

Supplementary Table S3. Oligonucleotides used in this study.

Supplementary Table S4. The growth data collected in this study.
